# Health Risk Assessment of Mercury Exposure from Fish Consumption in Munduruku Indigenous Communities in the Brazilian Amazon

**DOI:** 10.3390/ijerph18157940

**Published:** 2021-07-27

**Authors:** Ana Claudia Santiago de Vasconcellos, Gustavo Hallwass, Jaqueline Gato Bezerra, Angélico Nonato Serrão Aciole, Heloisa Nascimento de Moura Meneses, Marcelo de Oliveira Lima, Iracina Maura de Jesus, Sandra de Souza Hacon, Paulo Cesar Basta

**Affiliations:** 1 Laboratory of Professional Education in Health Surveillance, Joaquim Venâncio Polytechnic School of Health, Oswaldo Cruz Foundation, 21040-900 Rio de Janeiro, Brazil; 2 Laboratory of Human Ecology, Fish, Fisheries and Conservation, Postgraduate Program in Biosciences, Federal University of West Para, 68270-000 Oriximiná, Brazil; gustavo.hallwass@gmail.com (G.H.); jaqlinegb@gmail.com (J.G.B.); angelico.aciole@bol.com.br (A.N.S.A.); 3 Laboratory of Molecular Epidemiology, Postgraduate Program in Health Sciences, Federal University of West Para, 68040-470 Santarém, Brazil; heloisa.meneses@ufopa.edu.br; 4 Environment Section, Evandro Chagas Institute, Health Surveillance Secretariat, Ministry of Health, 67030-000 Ananindeua, Brazil; marcelolima@iec.gov.br (M.O.L.); iracinajesus@iec.gov.br (I.M.J.); 5 Samuel Pessoa Department of Endemics, National School of Public Health, Oswaldo Cruz Foundation, 21041-210 Rio de Janeiro, Brazil; sandrahacon@gmail.com

**Keywords:** mercury, indigenous, health risk assessment, Munduruku, fish, Brazilian Amazon

## Abstract

Fish serves as the principal source of animal protein for the indigenous people of the Amazon, ensuring their food and nutritional security. However, gold mining causes mercury (Hg) contamination in fish, and consequently increases health risks associated with fish consumption. The aim of this study was to assess the health risk attributed to the consumption of mercury-contaminated fish by Munduruku indigenous communities in the Middle-Tapajós Region. Different fish species were collected in the *Sawré Muybu* Indigenous Land to determine mercury levels. The health risk assessment was carried out according to the World Health Organization (WHO 2008) methodology and different scenarios were built for counterfactual analysis. Eighty-eight fish specimens from 17 species and four trophic levels were analyzed. Estimates of Hg ingestion indicated that the methylmercury daily intake exceeds the U.S. EPA (United States Environmental Protection Agency) (2000) reference dose from 3 to 25-fold, and up to 11 times the FAO (Food and Agriculture Organization)/WHO (2003) dose recommendation. In all situations analyzed, the risk ratio estimates were above 1.0, meaning that the investigated Munduruku communities are at serious risk of harm as a result of ingestion of mercury-contaminated fish. These results indicate that, at present, fish consumption is not safe for this Munduruku population. This hazardous situation threatens the survival of this indigenous population, their food security, and their culture.

## 1. Introduction

Fish has an important role in food security, since 17% of all animal protein consumed in the world is provided by fish [1]. In addition, fish consumption contributes to nutritional security, given its high content of essential nutrients (i.e., vitamins and minerals) and polyunsaturated fatty acids (e.g., omega-3 and omega-6) [2,3]. The food and nutritional security attributed to fish consumption is especially important for low-income populations in developing countries where over 90% of inland water caught fish are directed for local human consumption [4,5]. Indeed, fish is often the only quality protein accessible to poor people [6,7]. These reasons make fish a vital element for Amazonian populations, especially for indigenous and riverine populations.

The Amazon is the largest freshwater ecosystem and displays the greatest diversity of freshwater fish in the world [8,9]. Therefore, it is not surprising that Amazonian people have one of the highest fish consumption rates in the world [10,11,12]. An archaeological study found that fish was the species of vertebrates most consumed (>75% of total) in an ancient settlement of indigenous populations in the Central Brazilian Amazon. Fish consumption is historically related to culture and to food security of the original peoples from the Amazon [13,14]. However, several anthropogenic activities threaten the survival of Amazonian peoples, such as deforestation promoted by agribusiness (e.g., soybean cultivate and cattle), construction of hydroelectric dams, and artisanal gold mining [15,16,17,18,19,20,21,22]. The indigenous populations are particularly affected by the impact of these activities because they live in socially and environmentally vulnerable conditions caused by historical government neglect.

In this sense, the artisanal gold mining (also called *garimpos*) can be considered one of the most harmful economic activities in the Amazon, because it causes not only deforestation, river siltation, and soil erosion, but also releases large amounts of mercury into the environment. Mercury is a toxic heavy metal that contaminates the atmosphere, waters, sediments, and organisms [23,24].

Nevertheless, the current policies of the Brazilian federal government aim to allow oil and natural gas extraction, agribusiness, mining, and other economic activities in protected areas and indigenous lands. These policies are responsible for the highest rate of deforestation in the past 10 years, weakening the environmental protection legislation and human rights of traditional and indigenous populations [20,25,26].

In addition, recent studies carried out in the Amazon region indicate that illegal gold mining has had a sharp increase in the past years due to incentives from the federal government, mainly due to Bill 191/2020 presented by President Jair Bolsonaro to Parliament. The situation becomes even more serious because this increase is largely concentrated in indigenous areas, mainly affecting the Yanomami and Munduruku traditional territories [20,25].

The mercury used in *garimpos* is converted into methylmercury (MeHg), the most dangerous mercurial form to human health. Methylmercury undergoes bioaccumulation and biomagnification through aquatic trophic chains and, consequently, the consumption of contaminated fish and other organisms (e.g., crabs, shrimp, turtle, etc.) provides the main route of human exposure to this persistent environmental contaminant [27]. Ingested methylmercury is rapidly absorbed by the human gastrointestinal tract, principally affecting the central nervous and cardiovascular systems [28,29,30]. This mercurial form is especially harmful to pregnant women because the fetal brain is more sensitive to the action of methylmercury, causing many neurodevelopment problems to occur, including mental retardation, learning delays, visual and auditory alterations, and other harmful effects [31,32,33].

Considering this critical situation of mercury contamination within the Amazon and the consequences of human exposure to this contaminant, the present study aimed to assess the health risks from consumption of fish by mercury-exposed Munduruku indigenous communities in the Middle-Tapajós Region, in the state of Pará, one of the areas most threatened by illegal mining in the Brazilian Amazon. Risk assessment studies are of fundamental importance to identify population groups with a higher risk of exposure to a certain contaminant and can be the basis for the development of public policies to mitigate contamination.

## 2. Materials and Methods

### 2.1. Study Area

The present study was developed in the *Sawré Muybu* Indigenous Land (also known as Pimental), where a proportion of the Munduruku indigenous people live. This indigenous land is in the municipalities of Itaituba and Trairão, in the state of Pará, Brazil. The data collection and the fish capture were carried out between 29 October and 9 November 2019, in the villages *Poxo Muybu*, *Sawré Aboy*, and *Sawré Muybu* (see Basta et al. [34] to access the map and more details).

### 2.2. Fish Capture

The fish samplings were conducted in the mornings (8:00 to 12:00 a.m.) for seven consecutive days. All fish catches were conducted by the indigenous Munduruku themselves using their own fishing gears (i.e., gillnets and handlines) accompanied by field researchers. Indigenous fishermen employed gillnets with different sized mesh, one of them with 25 mm between opposite knots to catch bait to handline and other gillnets with 35 and 45 mm between opposite knots aiming to catch their target fish species. All fish caught were measured to standard length (cm), weighed (g), and the popular names recorded by researchers. Each fish specimen caught was identified to a species level in the field with further analyses in the laboratory, and the trophic level was recorded based on Santos et al. [35,36]. After the measurement and identification of the fish species, samples of 2 to 5 g of dorsal muscle tissue without skin or scales were collected from each specimen and stored in liquid nitrogen.

### 2.3. Mercury Analysis

The total mercury (THg) determined in the fish muscle tissue was obtained using the methodology proposed by Akagi et al. [37]. For each sample, 0.3 to 0.5 g of muscle tissue was weighed (wet weight) in a 50 mL Pyrex^®^ volumetric flask (Corelle Brands, Charleroi, Belgium). Then, 1 mL of deionized water, 2 mL of HNO_3_ and HClO_4_ (1:1), and 5 mL of H_2_SO_4_ were added for digestion. The vials were exposed to a hot plate (200 to 230 °C) for 30 min. After cooling to room temperature, the flasks were measured with deionized water and the digested samples were homogenized. The THg determination was made by cold vapor atomic absorption system (CVAAS), using semi-automatic mercury analyzer equipment Analyzer Model Hg-201 (Sanso Seisakusho Co. Ltd., Tokyo, Japan) [38]. To guarantee the Quality Assurance (QA)/ Quality Control (QC), we used for the mercury analysis in fish samples the following parameters: (i) reference materials dogfish liver certified reference material for trace metals (DOLT-4) (% of recovery: 92.24 ± 7.73; 70.92 to 100) and fish protein certified reference material for trace metals (DORM-3) (% of recovery: 96.22 ± 4.69; 87.16 to 100) from the National Research Council of Canada; (ii) a method blank; (iii) a 6-point calibration curve; and (iv) the relative standard deviation (RSD) of 8.32%. The detection and quantification limits (LOD/LOQ) obtained were 0.0083 ng/mg and 0.027 ng/mg, respectively.

Based on chemical analysis results, the mercury potential for biomagnification (between different trophic levels) and bioaccumulation (between different fish sizes of the same trophic level) was evaluated. The mercury biomagnification between four trophic levels sampled (i.e., piscivorous, omnivorous, herbivorous, and detritivorous) was checked by Kruskal–Wallis tests (residuals were not normal and variance non-homogeneous), with a post-hoc Dunn test. Bioaccumulation was analyzed using linear regression between THg levels and different fish sizes (standard length in cm) inside each trophic level. The linear regression was run due to expected relation of cause–effect between fish size and THg level. The residuals of regressions were checked, and one outlier, an individual of Piranha-preta (*Serrasalmus rhombeus*), was excluded from the analysis to ensure normality of the data and homogeneity of variances.

### 2.4. Data Collection: Participants’ Weight, Family Composition and Fish Consumption

To perform the health risk assessment related to consumption of mercury-contaminated fish, it was necessary to collect data about participants’ average weight (i.e., women, men, and children), fish consumption by Munduruku indigenous families (i.e., most consumed species and frequency), and family composition (i.e., number of individuals, age, and gender). The access to the amount of fish consumed (in grams) by the family members is described in the next section.

Data about diet were obtained through interviews with the head of households (husband/father), followed by weight measurements of all individuals living in the three investigated villages. The interview answers were recorded on electronic forms with the aid of portable devices (i.e., tablets). To measure weight, a portable digital scale from Seca^®^ (model 877) (Seca GmbH, Hamburg, Germany) was used, with a maximum capacity of 150 kg and precision of 0.1 kg.

### 2.5. Potential Fish Consumption Estimative from Catch Effort

The average amount of fish captured in one fishing day by the Munduruku indigenous was used to estimate the fish consumption by a family.

For the calculation of the family members’ fish consumption, we assumed as a premise that the effort of the Munduruku fishermen to catch fish during this fieldwork was similar to the fishing effort usually devoted by the heads of households. Due to this assumption, the average quantity of fish obtained could be a proxy for the quantity of fish available at home to feed a family for a week.

Taking into consideration that the amount of fish consumed varies according to the gender and age of the family member, we assumed that adult men consume 45% of the fish captured, adult women consume 35%, children aged 5 to 12 years consume 15%, while children from 2 to 5 years old consume only 5%.

### 2.6. Health Risk Assessment

The health risk assessment was carried out according to the methodology proposed by World Health Organization (WHO) [39]. We made two assumptions to calculate the daily mercury intake: (i) 100% of the mercury detected in the fish sample is in the form of methylmercury; (ii) 100% of the methylmercury available in the fish muscle tissue is absorbed in the human’s gastrointestinal tract. The amount of mercury ingested was estimated from the equation:

(1) $$\mathrm{MI}=\frac{\mathrm{FI}\times \mathrm{MC}}{\mathrm{KBW}}$$

where MI is methylmercury intake per kilogram body weight per day (µg methylmercury per kg body weight per day); FI is amount of fish ingested per day (g/day); MC is mercury concentration in the fish ingested (µg/g); KBW is kilogram body weight (kg bw).

We created different scenarios of methylmercury exposure from the data collected: (1) Rainy Season, (2) Dry Season, (3) Current, and (4) Critical. Scenarios 1 and 2 were constructed from the average mercury levels detected in fish species most consumed by the indigenous in the different seasons of the year, based on interview data. Scenario 3 was constructed from the weighted average of medium mercury concentrations detected in piscivorous and non-piscivorous species. The percentages of piscivorous and non-piscivorous species caught by the fishermen (34% and 66%, respectively) were multiplied by the average mercury levels detected. Scenario 4 was constructed from the 95th percentile of mercury concentrations in piscivorous and non-piscivorous species (1.42 and 0.29 µg/g, respectively). The 95th percentile was multiplied by the occurrence of the fish species, similarly to the previous scenario.

The risk ratio was calculated from the ratio between “the methylmercury estimated intake” and the reference doses proposed by U.S. EPA (United States Environmental Protection Agency) [40] and by FAO (Food and Agriculture Organization)/WHO [41]. According to U.S. EPA, the safe daily intake, also known as Reference Dose (RfD), is equal to 0.1 µg Hg/Kg bw/day. With the same purpose, FAO/WHO limits of 0.23 µg Hg/Kg bw/day for childbearing age women and for children and 0.45 µg Hg/Kg bw/day for adults in general.

When the risk ratio is less than 1, the risk of exposure is below the reference levels and, consequently, the risk of becoming ill is low. On the other hand, when the risk ratio is equal to or greater than 1, the risk of becoming ill due to mercury exposure must be considered. Therefore, the higher the risk ratio, the greater the risk of becoming ill due to mercury exposure.

## 3. Results

### 3.1. Fish Catch and Mercury Contamination

**Figure 1:**
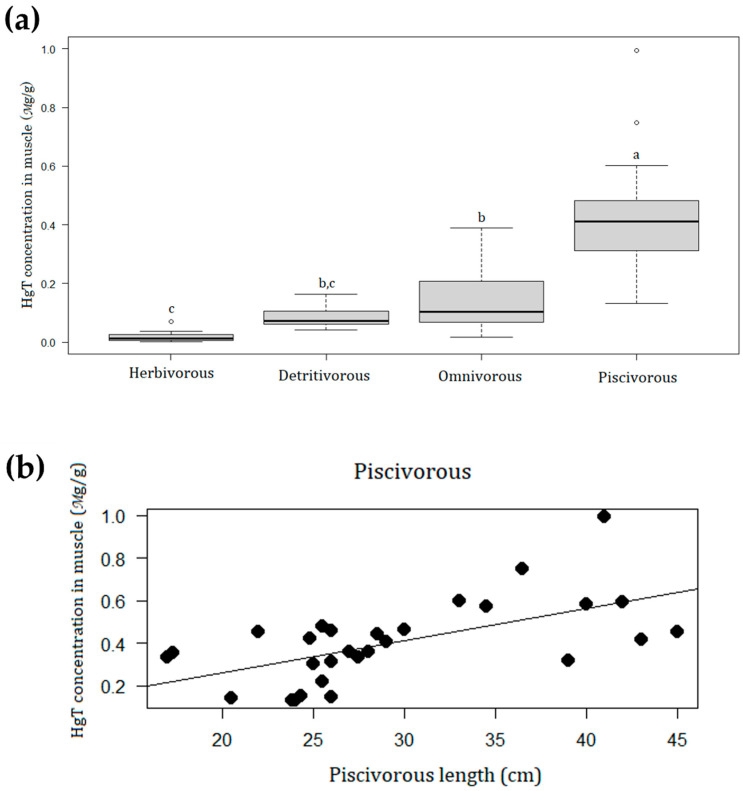
(**a**) Concentrations of mercury total (µg/g) in the muscle tissue of fish sampled (*n* = 88) compared among different trophic levels. Median (darker line in the box plot), minimum and maximum values (vertical lines), and outer lines of boxplot (25% and 75%). Dunn test: a > b > c, *p* < 0.05. Circles are outliers. (**b**) Linear regression between standard length (cm) of piscivorous fish (*n* = 29) and the concentration of mercury total (µg/g) in the muscle tissue of fish (y = −0.041 + 0.0151X; R^2^ = 0.36; *F* = 15.4; df = 27; *p* = 0.0005). One individual of Piranha-preta (*Serrasalmus rhombeus*) was excluded from the analysis to maintain normality and homogeneity of variances.

In total, 88 fish specimens were captured, distributed across 17 species and four trophic levels, as described in Table 1 showing the general characterization of the fish caught by the Munduruku fishermen during the fieldwork. Three piscivorous species showed average mercury levels above 0.5 µg/g. The biomagnification in the trophic chain of fish was confirmed, as there was a significant difference in the concentration of total mercury in muscle tissues among trophic levels (H = 60.2; df = 3; *p* < 0.0001) (Figure 1a). The average mercury levels in samples of non-piscivorous fish (*n* = 57) was 0.10 µg/g (SD = 0.09) and the average for piscivorous fish (*n* = 31) was 0.44 µg/g (SD = 0.34). In addition, bioaccumulation was found only in piscivorous species, where there was a positive and significant relationship between the size of the fish and the THg concentration in the muscle tissues (y = −0.041 + 0.0151X; R^2^ = 0.36; F = 15.4; df = 27; *p* = 0.0005) (Figure 1b). On the other hand, among other analyzed trophic levels (i.e., omnivorous, detritivorous, and herbivorous), there was no significant relationship between size and concentration of mercury (*p* > 0.05).

### 3.2. Weight Measurement, Family Composition and Fish Consumption

During the fieldwork, our team visited 35 domiciles: 20 in the *Sawré Muybu* village, 8 in the *Poxo Muybu* village, and 7 in the *Sawré Aboy* village. Among the participants, 53 were women of childbearing age (12 to 49 years old), 58 were adult men (≥12 years old), 24 were children aged 5 to 12 years old, and 42 were children aged 2 to 5 years. The interviews revealed that families are composed on average of 4 members (i.e., two adults and two children). According to the data collected in the fieldwork, the women of childbearing age had an average weight of 49.89 kg, adult men were 56.45 kg, children over 5 years old were 24.45 kg, and children under 5 years old were 14.07 kg (Table 2).

The questions about fish consumption revealed that 96% of the families consume fish regularly (≥3 times a week) and the most consumed species varied according to the season. During the rainy season, the most consumed species, in order of frequency related, are *surubim* (*Pseudoplatystoma* spp.), *barbado* (*Pinirampus pirinampu*), *aracu* (family Anostomidae), *tucunaré* (*Cichla* spp.), and *caratinga* (*Geophagus* spp.). The species most consumed in the dry season are *caratinga*, *curimatá* (*Prochilodus nigricans*), *surubim*, *pacu* (family Serrasalmidae), and *barbado*.

The average daily fish consumption estimates for the studied groups were the following: adult men consume 216.75 g (corresponding to 45% of the fish available for consumption), childbearing age women consume 168.58 g (35%), children over 5 years consume 72.25 g (15%), and children under 5 years consume 24.08 g (5%) (Table 2).

### 3.3. Health Risk Assessment

The analysis of the estimates reveals that the daily intake of methylmercury exceeds the reference limits recommended by the U.S. EPA [40] and FAO/WHO [41] in all scenarios built and in all studied population strata (Table 3). It means that in all hypothetical situations created in this study, the risk ratio estimates have values greater than 1.0. In summary, the Munduruku indigenous people living in the Middle-Tapajos River are at high risk of illness by the ingestion of mercury-contaminated fish. We can say that the less alarming risk ratio estimates (between 1.0 to 2.0) were observed in the population stratum represented by children aged 2 to 5 years, and by adults in general. Considering the safe dose proposed by FAO/WHO, risk ratio estimates under 2.0 were observed in children under 5 years old and adults in all hypothetical situations, except for the critical scenario. The risk ratio estimate for the critical scenario was 5.0 and 5.75 in children and adults, respectively (Table 3).

In our opinion, the current scenario is the closest to the reality of fish consumption by the Munduruku indigenous villages, and it indicates that all population segments ingest mercury in quantities above what is considered acceptable or safe. According to the U.S. EPA, women of childbearing age, who represent the most vulnerable demographic group to the effects of methylmercury, ingest 7 times more mercury than the reference dose proposed by this agency. According to FAO/WHO, the ingestion is 3 times higher than the safe intake. The critical scenario represents the levels of exposure observed in approximately 5% of the Munduruku population. In this case, women of childbearing age ingest 10 times more mercury than the limit proposed by the FAO/WHO, and 23 times more mercury than the safe limit proposed by the U.S. EPA. The analysis of the mercury levels in hair of the Munduruku population revealed that, in fact, there are individuals which presented mercury levels above 20 µg/g (more details in Basta et al. [34]).

## 4. Discussion

Fish are not only an essential source of protein, but many species are also rich in polyunsaturated fatty acids that reduce cholesterol levels in the blood, reduce the risk of myocardial infarction, and promote cognitive development [42,43]. Some authors point out that the annual average consumption of fish is 23 kg per capita in the Brazilian Amazon [44]. Frequently, the fish intake of riverside communities exceeds 300 g per day, resulting in annual average consumption that could surpass 100 kg per capita [10,45,46].

Despite the undeniable nutritional potential of fish, contaminants such as methylmercury has provoked significant debate about the balance between risks and benefits associated with fish consumption. Hu et al. [47] in a meta-analysis suggest that hair mercury concentration of 2–3 µg/g might be considered as a threshold for risk of developing hypertension. Fillion et al. [30] investigated riverine communities in the Amazon and showed an odds ratio equal to 2.91 (CI 95% 1.26–7.28) for elevated systolic blood pressure among individuals with hair Hg levels above 10 µg/g. In addition to these studies, Salonen et al. [29], in a longitudinal study with Finnish men, concluded that hair mercury levels above 2.0 µg/g represent a risk 69% higher for an acute myocardial infarction. Besides that, the neurotoxic effects of methylmercury have been known for a long time, since the Minamata tragedy in the 1950s and 1960s. The cohort studies conducted in the Faroe Islands and New Zealand indicate that even in low doses, the consumption of mercury-contaminated fish during pregnancy can cause important cognitive alterations in children [48,49]. The mercury neurotoxic potential effects in children and adults of the Amazon have been reported in recent publications [50,51,52,53,54]. The most common effects in children are cognitive problems, neurodevelopmental impairment, and psychomotor disorders. In adults, decreased visual field, neurobehavioral, and motor coordination disorders are most frequently reported [27].

Given the current federal government’s effort to create strategies to facilitate the invasion of protected areas in the Amazon by *garimpeiros* and mining industries, it is essential to clarify that the contamination of fish by mercury and all related health damages are caused (or intensified) by exploitation of gold. Many studies have already shown mercury contamination in the fauna of the Tapajós River Basin at least two decades ago [55,56,57,58,59,60,61,62,63,64,65]. Dórea et al. [59] detected mean mercury levels in piscivorous fish of 0.578 µg/g and 0.052 µg/g Hg in non-piscivorous in the upper Tapajós basin, whilst Brabo et al. [56] investigated fish contamination in the Sai Cinza region, also inhabited by indigenous Munduruku. They observed that the piscivorous species had mean mercury levels of 0.293 µg/g, while the non-piscivorous species had average mercury levels equal to 0.112 µg/g. These studies corroborate the present results that the mean mercury levels detected in piscivorous species are about 4 times greater than non-piscivorous species, highlighting the methylmercury biomagnification. Indeed, piscivorous fish were the only trophic level where we found a positive relationship between fish size and total Hg concentration, indicating bioaccumulation. Therefore, the larger the size of piscivorous fish, the higher the mercury concentration in their tissues and thus the higher the health risk for people who eat the larger ones.

In Brazil, the National Health Surveillance Agency (ANVISA) establishes the maximum concentrations of mercury in fish tissues that are judged appropriate for commercialization. Resolution No. 42 establishes that the maximum limit for inorganic mercury in fish is 0.5 µg/g for non-predatory species and 1.0 µg/g for predatory species [66]. Reflection on the applicability of these concentration limits promotes questions. The first question is, why propose limits for inorganic-mercury species, when almost all mercury present in the fish muscle is methylmercury, an organic mercury form? The second question is, how effective is the use of mercury concentration limits in fish in protecting the health of the population that consumes this fish?

We believe that the National Health Surveillance Agency (ANVISA) resolution does not promote any regulatory or normative effects, since the limits proposed by this agency are basically a wrong adaptation of the limits recommended by FAO/WHO [67] (0.5 µg MeHg/g for non-piscivorous fish and 1.0 µg MeHg/g for piscivorous fish). Furthermore, it is vital to clarify that these limits established by the FAO/WHO were adopted in 1991 and do not consider health effects produced by the ingestion of methylmercury in the fish [67]. The calculations for defining these maximum limits were performed based on data of the average mercury levels in fish samples of different trophic levels. Unfortunately, these values are frequently cited as safe levels for consumption.

The people’s diet is an extremely important cultural characteristic, as well as language and spiritualistic rituals. In indigenous communities, the inclusion of fish and other aquatic organisms as a diet items and the consumption frequency vary considerably among the groups living in the Amazon. The consumption of these items depends not only on the availability in the environment but also on the individual’s preferences as well as cultural patterns. For example, the Yanomami people who live in the Auaris region in the extreme northwest of Roraima state, as well as the Yanomami living in Venezuela, rarely eat fish [68,69]. On the other hand, fish consumption among Munduruku indigenous people can be considered high (at least three meals per day), varying slightly between different groups [56,58,59]. Studies that focus on the characterization of indigenous people’s diet face numerous difficulties, ranging from cultural and linguistic barriers to the choice of an effective method for quantifying the consumption of certain foods. Memory-based methods (recall method) about what was consumed by the family in the past 24 h or in the past few days generally produce over or underestimated data that rarely translate into reality and cause errors in estimates. Taking into consideration all the aspects exposed previously, it is extremely hard to measure during a study’s fieldwork the quantity in grams of fish consumed by each person in a day and the number of daily meals that include fish (and other aquatic organisms such as fish, crabs, mollusks, shrimp, turtles, etc.). From this point of view, the present study proposed a methodology for estimating potential fish consumption based on the catching of fish by indigenous themselves and then recorded by field researchers. The time devoted to fishing and the catching strategies were defined based on the reports of Munduruku fishermen.

With this challenge to estimate the food intake in culturally differentiated communities in mind, the goal of this study was to simulate an ordinary fishing day for the head of a Munduruku household. Thus, the mean amount of fish caught on a typical fishing day represents the amount of fish that a Munduruku family consumes over a week. Since there is no electricity in the homes visited, neither a refrigerator nor any other way of preserving food, the “moquém” technique (which is a type of smoking) is used to conserve fish.

To assess the accuracy of the empirical methodology accomplished in this investigation for estimating fish consumption, the daily mercury intake doses calculated for the current scenario were compared to the mercury levels detected in hair samples of the Munduruku indigenous people in the studied communities (data available in Basta et al. [34]). The current scenario was built to represent the mercury exposure scenario that most closely matches the local reality. The estimated mercury daily intake in this scenario for adult men and women was 0.828 and 0.729 µg/bw kg, respectively. The mean mercury level in male hair was 8.83 µg/g (SD: 4.56) and in women of childbearing age, the mean hair mercury level was 7.71 µg/g (SD: 3.88). If we consider that the intake of 0.1 µg/bw kg/day corresponds to hair mercury levels of 1.0 µg/g [39], the calculation of the amount of mercury intake daily is well-matched with the mercurial concentration detected in hair samples. Children from 5 to 12 years old also had a mercury daily intake dose matching with the levels of mercury in hair. The intake dose was estimated at 0.64 µg/bw kg/day and the average level of mercury in hair was 7.62 µg/g (SD: 5.44). Only the population stratum constituted by children aged 2 to 5 years did not indicate compatibility between intake and concentration in hair. In this case, the daily intake was estimated at 0.37 µg/bw kg/day and the average concentration in hair was 6.68 µg/g (SD: 3.44). Most likely, the difference found may be due to the influence of other routes of exposure, besides to fish consumption, such as breastfeeding and remnants of intrauterine transfer. However, there is a possibility that fish consumption was underestimated by our team for this age group.

The risk ratio estimated in this study indicates that there is no safe consumption of fish by the Munduruku population in any of the scenarios created for counterfactual analysis. Comparing the mercury intake doses in the different scenarios, we observed that mercury ingestion during the rainy season is higher than in the dry season. This result reflects the mercury levels detected in the most consumed fish species in this season of the year, according to reports by the study participants. In the rainy season, according to the interview reports, 60% of fish most consumed by the Munduruku families are piscivorous and therefore have higher levels of mercury.

According to the safety parameters proposed by FAO/WHO [41] and U.S. EPA [40], the entire study population is at risk of becoming ill due to the consumption of methylmercury contaminated fish. It is important to remember that the safe intake doses proposed by these international agencies were calculated from data produced in longitudinal studies. The dose of FAO/WHO [41] (PTWI: 1.6 µg MeHg/Kg bw/week) was derived from data produced in Seychelles, Faroe Islands, and New Zealand cohort studies. The dose recommended by U.S. EPA [40] was based only on the findings of the Faroe Islands cohort. However, the longitudinal studies mentioned above considered populations that differ strongly from the Amazonian populations and, probably, present mercury exposure thresholds for toxic health outcomes quite different from the indigenous communities.

The native people of the Brazilian Amazon are neglected by the State, which often becomes evident from the difficulty in accessing health services, the lack of sewage sanitation, and the high prevalence of many infectious diseases and child stunting. Besides this, the Amazonian ecosystem sees several risk factors for human exposure to mercury alongside each other which combine to create a uniquely dangerous situation. These factors include the presence of natural mercury in the soil as well as the development of activities that significantly change the mercury biogeochemical cycle in the region (e.g., artisanal gold mining, industrial gold mining, construction of dams and hydroelectric plants and agribusiness, which promotes forest burning and deforestation). This becomes evident that the development of a longitudinal study involving different population groups in the Amazon, such as indigenous, riverine, and urban populations, is especially important. Only after a long-term study will it be possible to estimate safe doses of mercury intake for the Amazonian population.

## 5. Conclusions

The current gold mining activity in the Middle-Tapajós Region is causing environmental devastation, social conflict, and increasing mercury levels in the environment. This activity causes mercury accumulation in fish, especially in piscivorous. Consequently, the population living in this region consumes contaminated fish and compromises their health. The present study revealed that the fish collected in the rivers that cross the *Sawré Muybu* Indigenous Land have mercury concentrations with the potential to harm the health of the Munduruku population, particularly women of childbearing age and children. In all of the scenarios created for counterfactual analysis, the estimated risk ratios are greater than 1.0, indicating that the intake of mercury by the groups studied is higher than the limits proposed by health agencies. However, we highlight that fish is an important element of Munduruku culture and is an essential animal protein for riverside and indigenous populations across the Amazon. The mercury contamination observed in fish and the indigenous Munduruku is a direct consequence of gold mining and the Brazilian authorities’ longstanding refusal to condemn this activity, threatening the health and rights of the native peoples. Therefore, we refute the current policies of the Brazilian federal government regarding the permission of mining in Indigenous Land. In conclusion, we recommend the immediate closure of illegal gold mining in the Brazilian Amazon, the principal cause of mercury contamination in the region.

## Figures and Tables

**Table 1:** Characterization of the species of fish caught, *Sawré Muybu* Indigenous Land, Pará, Amazon, Brazil, 2019.

Fish Species	Popular Name	N	Size (cm)	Weight (g)	Trophic Level	Hg (µg/g) (SD)	Min–Max (Hg)
*Serrasalmus rhombeus*	Piranha Preta	6	17–34.5	140–1305	Piscivorous	0.71 (±0.61)	0.33–1.95
*Pseudoplatystoma fasciatum*	Surubim	6	23.8–45	141–907	Piscivorous	0.24 (±0.15)	0.13–0.45
*Pinirampus pirinampu*	Barbado	8	17.3–42	109–961	Piscivorous	0.49 (±0.14)	0.31–0.75
*Cichla ocellaris*	Tucunaré	6	25–29	347–571	Piscivorous	0.33 (±0.06)	0.22–0.41
*Rhaphiodon vulpinus*	Peixe Cachorro	2	39–41	328–469	Piscivorous	0.66 (±0.48)	0.32–1.00
*Ageneiosus inermis*	Mandubé	1	33	550	Piscivorous	0.6	-
*Pachyurus junki*	Corvina	1	20.5	148	Piscivorous	0.14	-
*Geophagus proximus*	Caratinga	10	10.5–18.5	36–171	Omnivorous	0.07 (±0.03)	0.03–0.10
*Pimelodus blochii*	Mandii	7	14.5–17.3	60–84	Omnivorous	0.20 (±0.05)	0.13–0.28
*Leporinus fasciatus*	Aracu Flamengo	5	17.7–23.3	102–244	Omnivorous	0.09 (±0.02)	0.05–0.11
*Caenotropus labyrinthicus*	João Duro	6	13.7–14.8	63–73	Omnivorous	0.28 (±0.07)	0.17–0.39
*Hemiodus unimaculatus*	Charuto	1	17.5	95	Omnivorous	0.02	-
*Schizodon vittatus*	Aracu	4	21.3–27.3	163–351	Herbivorous	0.03 (±0.01)	0.02–0.04
*Myloplus rubripinnis*	Pacu Branco	7	12.5–20.5	89–390	Herbivorous	0.02 (±0.03)	0.01–0.07
*Semaprochilodus insignis*	Jaraqui Escama Grossa	6	21–23.5	223–329	Detritivorous	0.11 (±0.05)	0.05–0.16
*Prochilodus nigricans*	Curimatá	6	20.5–24	253–369	Detritivorous	0.07 (±0.02)	0.04–0.10
*Curimata* sp.	Branquinha	6	12.7–14	64–85	Detritivorous	0.09 (±0.03)	0.06–0.13

**Table 2:** Data collected and estimates, *Sawré Muybu* Indigenous Land, Pará, Amazon, Brazil, 2019.

**Fish Catch**		
Total fish caught (*n*°)	88	
Catch period (days)	7	
Fish caught per day (*n*°)	12.6	
Average weight of fish (grams)	268.2	
Amount of fish per family (grams)	3371.7	
**Family composition and weight measurements (Kg)**		
Average number of individuals per family	4	
Average number of adults	2	
Average number of children	2	
Average weight of childbearing age women (*n* = 53)	49.89	
Average weight of adult men (≥12 years) (*n* = 58)	56.45	
Average weight of children (from 5|−12 years old) (*n* = 24)	24.45	
Average weight of children (2|−5 years) (*n* = 42)	14.07	
**Fish consumption estimative (grams)**		
	Weekly Intake	Daily Intake
Adult men (45%)	1517.2	216.75
Childbearing age women (35%)	1180.1	168.58
Children 5|−12 years old (15%)	505.7	72.25
Children aged 2|−5 years (5%)	168.6	24.08

**Table 3:** Estimated of methylmercury intake dose and risk ratio in different scenarios, *Sawré Muybu* Indigenous Land, Pará, Amazon, Brazil, 2019.

Scenarios Constructed	Hg-intake Dose µg/kg bw/day	Risk Ratio		
		U.S. EPA	FAO/WHO (Women and Children)	FAO/WHO (Adults in General)
Scenario 1—Rainy Season				
Women of childbearing age	0.78	7.84	3.41	N.A.
Adult Men	0.89	8.91	N.A.	1.98
Children 5|−12 years old	0.69	6.86	2.98	N.A.
Children 2|−5 years	0.40	3.97	1.73	N.A.
Scenario 2—Dry Season				
Women of childbearing age	0.59	5.95	2.59	N.A.
Adult Men	0.68	6.76	N.A.	1.50
Children 5|−12 years old	0.52	5.20	2.26	N.A.
Children 2|−5 years	0.30	3.01	1.31	N.A.
Scenario 3—Current				
Women of childbearing age	0.73	7.29	3.17	N.A.
Adult Men	0.83	8.28	N.A.	1.84
Children 5|−12 years old	0.64	6.37	2.77	N.A.
Children 2|−5 years	0.37	3.69	1.60	N.A.
Scenario 4—Critical				
Women of childbearing age	2.28	22.76	9.90	N.A.
Adult Men	2.59	25.86	N.A.	5.75
Children 5|−12 years old	1.99	19.90	8.65	N.A.
Children 2|−5 years	1.15	11.53	5.01	N.A.

U.S. EPA = United States Environmental Protection Agency; FAO = Food and Agriculture Organization; WHO = World Health Organization.

## Data Availability

Data sharing not applicable.
